# Genome-Wide Identification of the *TCP* Gene Family in *Broussonetia papyrifera* and Functional Analysis of *BpTCP8*, *14* and *19* in Shoot Branching

**DOI:** 10.3390/plants9101301

**Published:** 2020-10-01

**Authors:** Meiling Zhao, Xianjun Peng, Naizhi Chen, Shihua Shen

**Affiliations:** 1 Key Laboratory of Plant Resources, Institute of Botany, Chinese Academy of Sciences, Beijing 100093, China; zml@ibcas.ac.cn (M.Z.); pengxianjun@ibcas.ac.cn (X.P.); 2 University of Chinese Academy of Sciences, Beijing 100049, China

**Keywords:** *B. papyrifera*, *TCP* gene family, gene expression, shoot branching

## Abstract

The plant-specific TCP family proteins play an important role in the processes of plant growth and development. *Broussonetia papyrifera* is a versatile perennial deciduous tree, and its genome data have been published. However, no comprehensive analysis of the *TCP* gene family in *B. papyrifera* has been undertaken. In this study, 20 *BpTCP* genes (*BpTCPs*) were identified in the *B. papyrifera* genome. Phylogenetic analysis divided *BpTCPs* into three subclades, the PCF subclade, the CIN subclade and the CYC/TB1 subclade. Gene structure analysis displayed that all *BpTCPs* except *BpTCP19* contained one coding region. Conserved motif analysis showed that BpTCP proteins in the same subclade possessed similar motif structures. Segmental duplication was the primary driving force for the expansion of *BpTCPs*. Expression patterns showed that *BpTCPs* may play diverse biological functions in organ or tissue development. Transcriptional activation activity analysis of BpTCP8, BpTCP14 and BpTCP19 showed that they possessed transcriptional activation ability. The ectopic expression analysis in *Arabidopsis* wild-type and *AtBRC1* ortholog mutant showed that *BpTCP8*, *BpTCP14* and *BpTCP19* could prevent rosette branch outgrowth. Collectively, our study not only established the first genome-wide analysis of the *B. papyrifera TCP* gene family, but also provided valuable information for understanding the function of *BpTCPs* in shoot branching.

## 1. Introduction

The TCP family is a plant-specific transcription factor family, which was first found in 1999 [1]. The name of TCP transcription factor family was derived from the four proteins originally discovered, TEOSINTE BRANCHED 1 (TB1) from *Zea mays*, CYCLOIDEA (CYC) from *Antirrhinum majus* and the PROLIFERATING CELL FACTORS 1 and 2 (PCF 1 and PCF2) from *Oryza sativa* [2,3,4]. The protein sequences of TCP transcription factors contain a conserved non-canonical basic helix-loop-helix (bHLH) motif of about 59 amino acids, which was called the TCP domain. According to the differences of TCP domains, the TCP family members can be divided into two subfamilies: class I (composed of the PCF subclade) and class II (composed of the CIN and CYC/TB1 subclades) [5,6]. The most noteworthy difference between these two subfamilies is that the basic region of TCP domain of class II family has four amino acids more than that of class I family. In addition, several members of class II have another conserved region outside the TCP domain named the R domain, which is an arginine-rich motif containing eighteen to twenty residues [6]. The R domain is hypothesized to form a coiled coil that may be involved in protein–protein interactions [7].

Accumulating evidence demonstrate that *TCP* genes play a crucial part in many biological processes of plant growth and development, including leaf morphogenesis, leaf development, floral asymmetry, branching, seed germination, circadian rhythm, defense response and hormone signal transduction pathway [8,9,10,11,12,13,14,15,16,17,18,19,20,21,22,23,24]. Based on the finding of OsPCF1 and OsPCF2, members of class I are usually assumed to promote cell differentiation and plant growth [4]. Taking *Arabidopsis* as an example, *AtTCP14* and *AtTCP15* were involved in cell proliferation during the development of internode, leaf and seed [14,25]. *AtTCP7* played a major role in leaf and hypocotyls, redundant with *AtTCP8*, *AtTCP14*, *AtTCP15*, *AtTCP21*, *AtTCP22* and *AtTCP23*, due to the endoreplication defects, its dominant-negative mutant exhibited a phenotype with smaller leaf cells and shorter hypocotyls compared to the wild type [26]. *AtTCP16* is of great importance in pollen development and predominantly expressed in developing microspores. Its RNAi transgenic plants produced equal amounts of normal and abnormal pollen grains that gave rise to morphological abnormality and degeneration of genomic DNA [27]. *AtTCP9* and *AtTCP19* played a part in the control of leaf senescence in a redundant fashion with *AtTCP20*, which both of the double mutants *tcp9tcp20* and *tcp19tcp20* initiated the senescence program earlier than the wild type, but there was no performance in the single mutants [21,28]. In class II, genes of the CIN subclade may participate in regulating leaf morphogenesis. In *Arabidopsis*, *AtTCP2*, *AtTCP3*, *AtTCP4*, *AtTCP10* and *AtTCP24* that contain a microRNA (miRNA) *miR319* binding site have been proven that their quadruple mutant showed a phenotype of strongly crinkled leaves through up-regulating the cyclin genes and the genes relative with the cell division [9,29]. The other three genes of the CIN subclade, *AtTCP5*, *AtTCP13* and *AtTCP17*, redundantly have vital roles in promoting hypocotyl elongation by means of up-regulating auxin biosynthesis under the shade-induced conditions, and the elongation of the hypocotyls of their triple mutant was suppressed significantly [30,31]. It is commonly thought that CYC/TB1 subclade genes of class II are involved in the growth of lateral branch and floral symmetry on the basis of appearance of ZmTB1 and AmCYC [2,3]. In *Arabidopsis*, there are three genes in this subclade, *AtTCP1*, *AtTCP12* and *AtTCP18*. *AtTCP1* is the homolog of *AmCYC*, which positively regulated the brassinosteroid (BR) biosynthesis pathway via directly mediating the expression of the key BR biosynthetic gene *DWARF4* (*DWF4*) [23,32]. In addition, there is solid evidence that *AtTCP1* may directly or indirectly regulate several signal pathway known to be significant for plant growth and development [33]. *AtTCP18* (also called *BRANCHED1*, *BRC1*) and *AtTCP12* (also called *BRANCHED2*, *BRC2*) are closely related to *ZmTB1*. Both take part in suppressing bud outgrowth, but mainly *BRC1*; the expression of *BRC1* largely restricted to axillary buds and *brc1* mutant showed an exclusive regulation of the axillary bud outgrowth [11,34].

*Broussonetia papyrifera*, also known as paper mulberry, a common East Asian perennial deciduous tree, belongs to *Broussonetia* of Moraceae. It is widely distributed in the south of China, Japan, Korea, most of Southeast Asia countries and also in the countries of Oceania and the Americas [35]. *B. papyrifera* is a multifunction tree that is widely used in medicine, livestock feed, papermaking and ecological afforestation because of its various biological activities, high quality fiber properties and strong resistance ability [36,37,38,39]. Different plant types of *B. papyrifera* are needed to achieve diverse applications. In China, the branches and leaves of *B. papyrifera* are mainly used for silage. It is reported that diet with 10–15% *B. papyrifera* silage could enhance the antioxidant capacity and strengthen the immune system of dairy cows, while improving the quality of milk by increasing the polyunsaturated fatty acid concentration [40]. Thus, the number of branches is great importance for *B. papyrifera*, because the architecture with plenty branches and leaves is needed to ramp up production. To date, the *TCP* gene family has been identified in multiple species, for instance, *Arabidopsis*, *Oryza sativa* (rice), *Panicum virgatum* (switchgrass), *Vitis vinifera* (grape), *Z. mays* (maize), *Phaseolus vulgaris* (common bean) and *Citrullus lanatus* (watermelon) [6,41,42,43,44,45]. It is proved that *TCP* genes have important functions in shoot branching and plant growth. Hence, we carried out a global analysis of the *TCP* gene family in *B. papyrifera* according to the whole genome sequencing data [46]. In this study, 20 non-redundant *BpTCP* genes were identified and a systematic analysis was performed, including phylogenetic relationships, gene structure, conversed motifs, chromosomal location, duplication events and expression patterns in different tissues. Meanwhile, the functions of *BpTCP8*, *BpTCP14* and *BpTCP19* in regulating shoot branching were validated by ectopic expression in *Arabidopsis* wild-type and *AtBRC1* ortholog mutant.

## 2. Results

### 2.1. Identification and Property Analysis of the TCP Genes in B. papyrifera

To identify the *TCP* genes in *B. papyrifera*, local BLASTp and HMM searches were performed against the *B. papyrifera* genome database using 24 known *Arabidopsis* TCP protein sequences. Subsequently, the non-redundant candidate BpTCP protein sequences of the two methods were submitted to the NCBI CD Search and InterPro database for further verification. Finally, a total of 20 BpTCP proteins were identified, which contained the conserved TCP domain. According to the *B. papyrifera* genomic information, a chromosome location map was constructed to illustrate the distribution of the *BpTCP* genes on each chromosome. The map showed that all *BpTCP* genes were unevenly mapped onto 10 out of 13 *B. papyrifera* chromosomes and the *BpTCP* genes were annotated as *BpTCP1* to *BpTCP20* in the light of their physical locations on the chromosomes (Figure S1).

In order to understand the BpTCP proteins more comprehensively, the physical and chemical properties were analyzed (Table 1). The length of BpTCP proteins varied from 165 (BpTCP11) to 566 (BpTCP12) amino acid residues. The molecular weight (MW) ranged from 18,140.32 (BpTCP11) to 59,824.02 (BpTCP12) Da. The protein isoelectric point (pI) distributed from 5.26 (BpTCP9) to 9.72 (BpTCP20) with a mean of 7.501. All the BpTCP proteins were predicted to localize in the nucleus. The prediction of the phosphorylation site displayed that the BpTCP proteins contained 1 (BpTCP15) to 9 (BpTCP8) phosphorylation sites, except BpTCP9 and BpTCP11 that had no phosphorylation site. Among them, 17 BpTCP proteins contained Ser site with the number ranging from 1 to 8, and 11 BpTCP proteins contained Thr site with the number ranging from 1 to 3. Only BpTCP15 and BpTCP18 contained Tyr site with the number of 1 and 3, respectively.

### 2.2. Phylogenetic Relationship of BpTCP Proteins and Distribution of TCP Proteins in Plants

**Figure 1:**
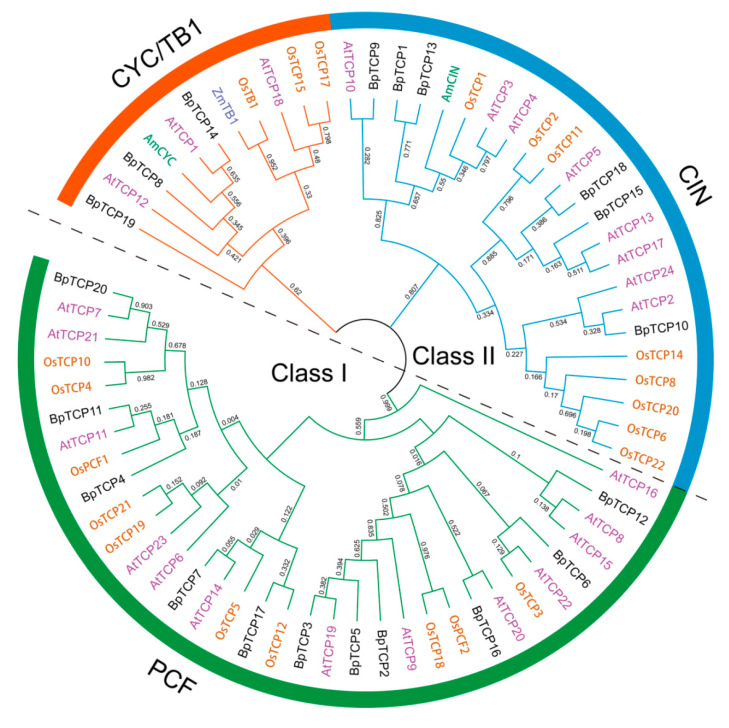
Phylogenetic analysis of TEOSINTE BRANCHED 1/CYCLOIDEA/PROLIFERATING CELL FACTORS 1 and 2 (TCP) proteins among *B. papyrifera*, *A. thaliana* and *O. sativa*. The full-length amino acid sequences of TCP proteins from *B. papyrifera*, *A. thaliana*, *O. sativa*, *Z. mays* (ZmTB1) and *A. majus* (AmCYC and AmCIN) were aligned and the phylogenetic tree was constructed by MEGA X using the maximum likelihood (ML) method with 1000 bootstrap replicates. The numbers on each branch line represent the bootstrap values. Different species are shown in different colored fonts. Subtree branches marked with different colors represent different subclades.

To comprehend the accurate classification of BpTCP proteins and the phylogenetic relationship with other known TCP proteins, we used the full length sequences of 65 TCP proteins from *B. papyrifera*, *Arabidopsis*, *O. sativa* and 3 TCP proteins (AmCIN, AmCYC and ZmTB1) with known functions to establish an unrooted phylogenetic tree through MEGA X software (Figure 1). The results showed that the BpTCP proteins were obviously classified into two subfamilies: class I (namely PCF subclade) and class II (contained CIN and CYC/TB1 subclade). Moreover, the TCP proteins of *Arabidopsis* and *O. sativa* exhibited the same classification as previous studies [6,47], which verified the reliability of the phylogenetic tree. In accordance with the classification, there were 11 BpTCP proteins in the PCF subclade, 6 BpTCP proteins in the CIN subclade and 3 BpTCP proteins in the CYC/TB1 subclade.

**Figure 2:**
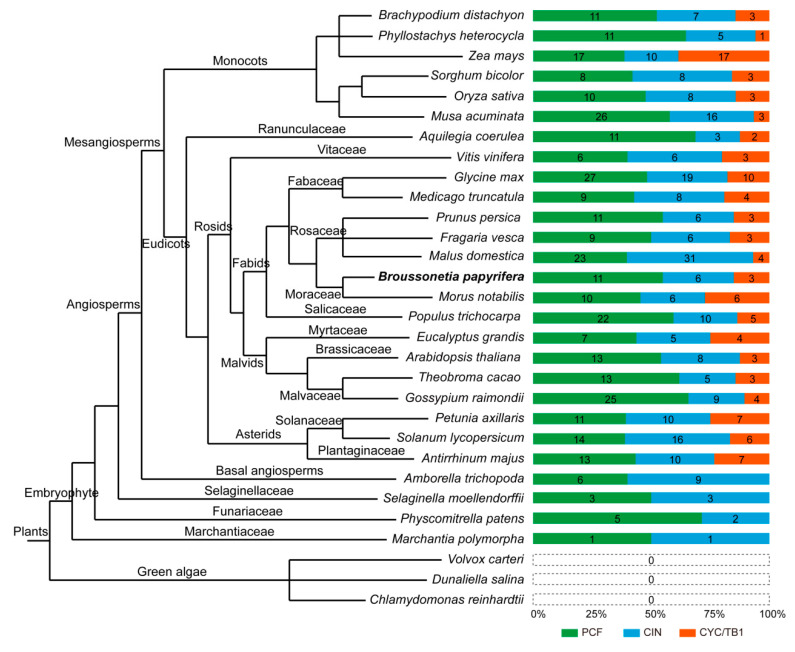
Distribution of TCP family members in plants. Each color of the chart on the right represents a subclade, and the numbers indicate the number of TCP members in the subclade.

Meanwhile, in order to understand the evolutionary relationship of TCP family among different plants, we analyzed the composition of TCP family on 30 species (including angiosperms, ferns, mosses and green algae) and the number of TCP proteins of each subclade in every species was annotated (Figure 2, Table S1). As the figure shows, there are no TCP proteins in *Volvox carteri*, *Dunaliella salina* and *Chlamydomonas reinhardtii*. Only a few TCP proteins exist in *Selaginella moellendorffii* (6), *Physcomitrella patens* (7), *Marchantia polymorpha* (2), and they do not contain CYC/TB1 subclade. Besides, the basal angiosperm *Amborella trichopoda*, which has radially symmetrical flowers, contains 15 TCP proteins and also lacks CYC/TB1 subclade. Both monocots and eudicots have the CYC/TB1 subclade, the number of CYC/TB1 subclade members varies from 1 to 17 and the number of the whole TCP family members varies greatly among species. The range of TCP proteins in monocots is 17 to 21, with exceptions of 44 for *Z. mays* and 45 for *Musa acuminate*, and the number of CYC/TB1 subclade members is 17 in *Z. mays* and 3 in *M. acuminate*, which is a significant difference. The number of TCP proteins in eudicots ranges from 15 (*Vitis vinifera*) to 58 (*Malus domestica*), among which the corresponding number of CYC/TB1 subclade members is 3 and 4. *B. papyrifera* and *Morus notabilis* belong to Moraceae and the number of TCP proteins is similar, which is 20 and 22, respectively. However, the number of CYC/TB1 subclade is 6 in *M. notabilis* and 3 in *B. papyrifera*. In general, the number of TCP members in different species varies substantially. The CYC/TB1 subclade appeared after the emergence of radially symmetrical flowers and the proportion of CYC/TB1 subclade members in the whole TCP family was not linearly related to the total number.

### 2.3. Analysis of Gene Structure and Conserved Motifs

**Figure 3:**
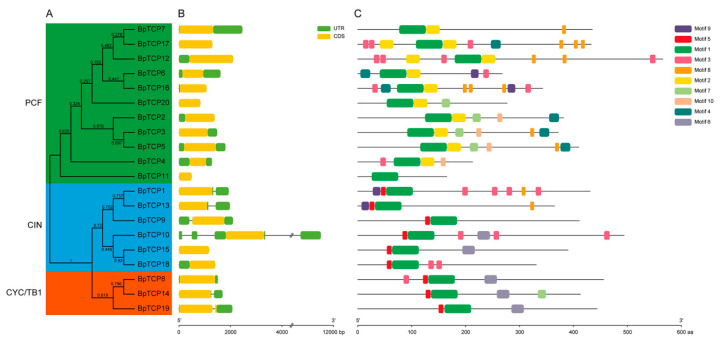
Phylogenetic relationship, gene structure and conserved motif analysis of the BpTCP family. (**A**) The conserved TCP domain amino acid sequences of BpTCP proteins were aligned and a ML tree was constructed by MEGA X with 1000 bootstrap replicates. The numbers on each branch line represent the bootstrap values. The colored blocks indicate different subclades. (**B**) The gene structure of *BpTCPs*. Exons, introns and untranslated regions (UTRs) are indicated by yellow rectangles, black lines and green rectangles, respectively. (**C**) The multiple conserved motifs of BpTCP proteins. Different colored rectangles represent different motifs.

To get a better insight into the evolutionary relationships and explore the structure diversification of the BpTCP family in each subclade, we constructed a new phylogentic tree of BpTCP proteins, and analyzed the gene structure and conserved motifs of BpTCP family. The phylogenetic tree of the BpTCP family was constructed with the conserved TCP domain sequences, and the result showed that it was also divided into three subclades (Figure 3A). The gene structures of *BpTCP* genes were remarkably similar (Figure 3B). There were 19 *BpTCPs* with one coding region and only *BpTCP19* that belongs to the CYC/TB1 subclade contained one intron in the coding region. Conserved motifs occupy an important role in the characteristic analysis and classification of gene family. Using the MEME online program, 10 motifs were identified among the BpTCP proteins (Figure 3C and Figure S2). As shown, BpTCP proteins in the same subfamilies possess similar motif composition. Motif 1 (TCP domain) exists in all BpTCP members. Motif 2 exists in all class I (PCF subclade) members (except BpTCP11). Motif 5 exists in all class II (CIN and CYC/TB1 subclade) members. In addition, motif 3 is distributed in 9 out of 20 BpTCP proteins, which are scattered throughout each subclade. Motif 4 and motif 10 are exclusively present in part of the PCF subclade members. Motif 6 is exclusively present in 2 CIN subclade members and all CYC/TB1 subclade members. Motif 7 exists in several PCF and CYC/TB1 subclade members. Motif 8 and motif 9 only exist in some PCF and CIN subclade members. These results suggest that the motifs exclusively existing in a certain subclade may be relative to the specific function.

### 2.4. Chromosome Location and Duplication Event Analysis

**Figure 4:**
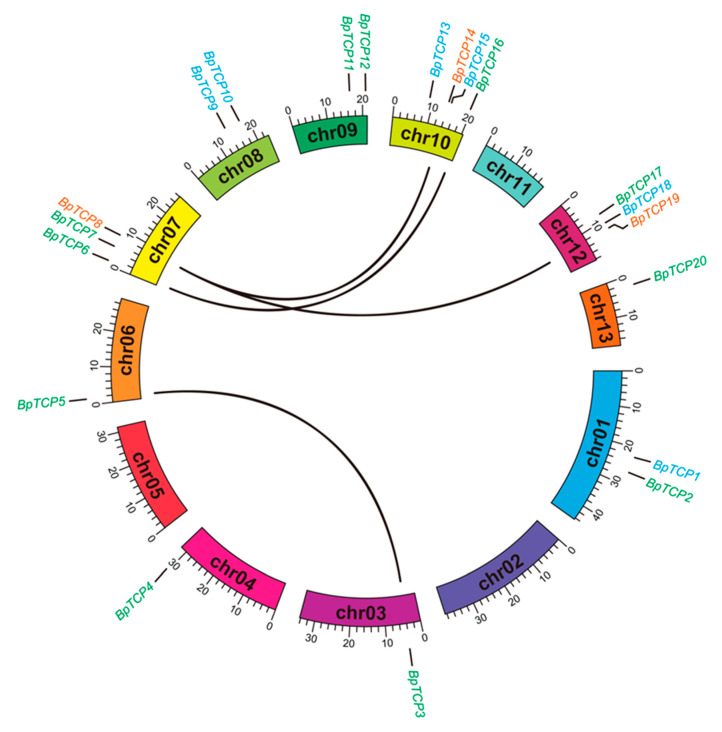
Chromosome location and duplication event analysis of *BpTCP* genes. The colored bands indicate *B. papyrifera* chromosomes. The gene names with different colors represent different subclades. Genes with duplication relationships are connected by black lines in the inner part.

Gene duplications are considered to play a vital part in the expansion and evolution of the gene family, so the duplication event analysis of *BpTCP* genes was performed (Figure 4). The results showed that there was no tandem duplication events observed, which suggested that segmental duplications may be the primary driving force for the expansion of BpTCP family. A total of four pairs of paralogous *BpTCP* genes were identified and they distributed on five chromosomes. Among these genes, four genes (*BpTCP3*, *BpTCP5*, *BpTCP6* and *BpTCP16*) belong to the CIN subclade and three genes (*BpTCP8*, *BpTCP14* and *BpTCP19*) belong to the CYC/TB1 subclade. The four segmental duplication events were *BpTCP3*/*BpTCP5*, *BpTCP6*/*BpTCP16*, *BpTCP8*/*BpTCP14* and *BpTCP8*/*BpTCP19*, respectively.

### 2.5. Expression Pattern Analysis of BpTCP Genes

**Figure 5:**
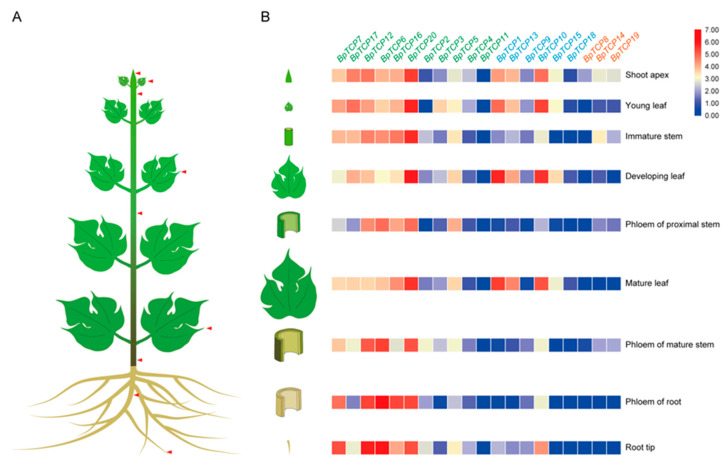
Expression patterns of *BpTCP* genes in different tissues. (**A**) Schematic drawing of tissue sampling. (**B**) Heat map of the expression patterns. Expression values were transformed to log2. The color scale represents relative expression levels.

To provide more insight into the potential roles of *BpTCP* genes during growth and development, the expression patterns of *BpTCP* genes were investigated based on the RNA-Seq data of nine tissues (shoot apex, young leaf, developing leaf, mature leaf, immature stem, phloem of proximal stem, phloem of mature stem, phloem of root and root tip) (Figure 5). As shown in the heat map, genes belonging to the same subclade did not always share similar expression patterns. Some genes possessed similar expression patterns in different tissues, while some genes exhibited significant tissue specificity. For example, among PCF subclade genes, *BpTCP5*, *BpTCP6*, *BpTCP7*, *BpTCP12*, *BpTCP16*, *BpTCP17* and *BpTCP20* were expressed in almost all tissues, whereas *BpTCP11* was hardly expressed in all tissues. *BpTCP2* and *BpTCP4* were lowly expressed in all tissues. The expression level of *BpTCP3* in young leaf was higher than other tissues. In the CIN subclade, *BpTCP1*, *BpTCP10*, *BpTCP13* and *BpTCP15* showed the similar expression patterns, which were highly expressed in shoot apex, young leaf, developing leaf and mature leaf. *BpTCP9* and *BpTCP18* were expressed at low level in all tissues. In the CYC/TB1 subclade, the expression levels of *BpTCP14* and *BpTCP19* were a little higher in shoot apex and immature stem than other seven tissues. *BpTCP8* was slightly expressed in the shoot apex and extremely lowly expressed in other eight tissues.

### 2.6. Quantitative RT-PCR of BpTCP8, BpTCP14 and BpTCP19

**Figure 6:**
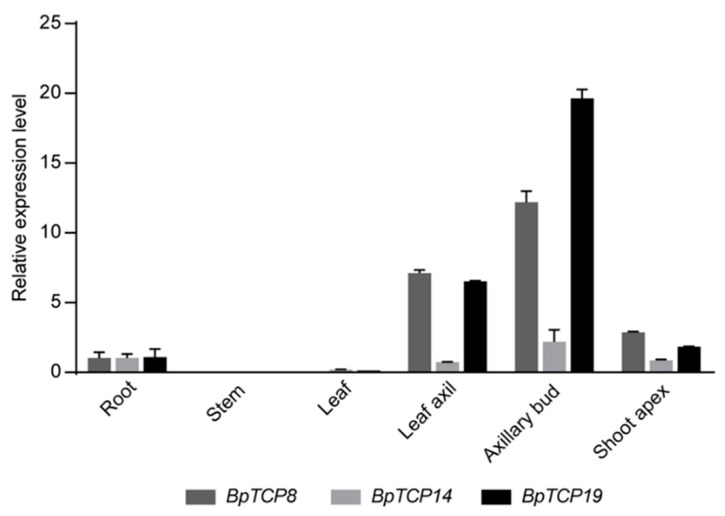
The expression levels of *BpTCP8*, *BpTCP14* and *BpTCP19* in different tissues detected by quantitative RT-PCR. Expression level of *BpGAPDH* was detected as an internal control. Error bars indicate standard deviation of three biological replications.

*AtBRC1* (*AtTCP18*) and *AtBRC2* (*AtTCP12*), which belong to the CYC/TB1 subclade, were demonstrated to regulate the shoot branching by arresting bud development [11]. In addition, plant architecture is of great significance for the application of *B. papyrifera*. Therefore, we turned our focus to the CYC/TB1 subclade of the BpTCP family. Quantitative RT-PCR was carried out to compare expression level of *BpTCP8*, *BpTCP14* and *BpTCP19* in shoot apex, leaf axil, axillary bud, leaf, stem and root (Figure 6). The results showed that *BpTCP8* and *BpTCP19* exhibited an extremely prominent expression level in leaf axil and axillary bud. *BpTCP14* exhibited higher expression level in axillary bud, but much lower than *BpTCP8* and *BpTCP19*.

### 2.7. Transcriptional Activation Activity of BpTCP8, BpTCP14 and BpTCP19

**Figure 7:**
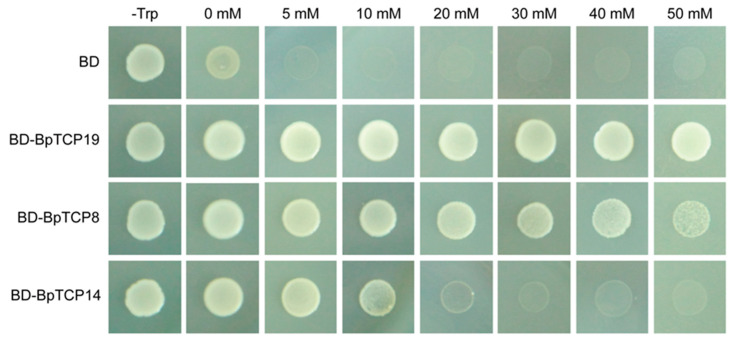
Transcriptional activation activity analysis of BpTCP8, BpTCP14 and BpTCP19. The full-length coding sequences of *BpTCP8*, *BpTCP14* and *BpTCP19* were fused into *p*Bridge, and the transformed AH109 strains were incubated in SD-Trp medium and SD-Trp-His medium supplemented with 0–50 mM 3-AT. The negative control was the empty *p*Bridge (BD) vector.

The transcriptional activation activity of BpTCP8, BpTCP14 and BpTCP19 was tested by yeast one-hybrid assay. The degree of transcriptional activation activity can be measured by observing the ability of transformed yeast cells growing on the selective medium with 0–50 mM HIS3 protein competitive inhibitor 3-aminotriazole (3-AT). All transformants of *BpTCP8*, *BpTCP14* and *BpTCP19* could readily grow on the SD-Trp medium (Figure 7). In the SD-Trp-His medium without 3-AT, only the yeast cells containing *BpTCPs* could grow well. In the SD-Trp-His medium supplemented with different concentrations of 3-AT, the yeast cells containing *BpTCPs* exhibited different growth capacity. BpTCP19 exhibited the strongest activation activity, followed by BpTCP8 and finally BpTCP14.

### 2.8. Roles of BpTCP8, BpTCP14 and BpTCP19 in Shoot Branching

**Figure 8:**
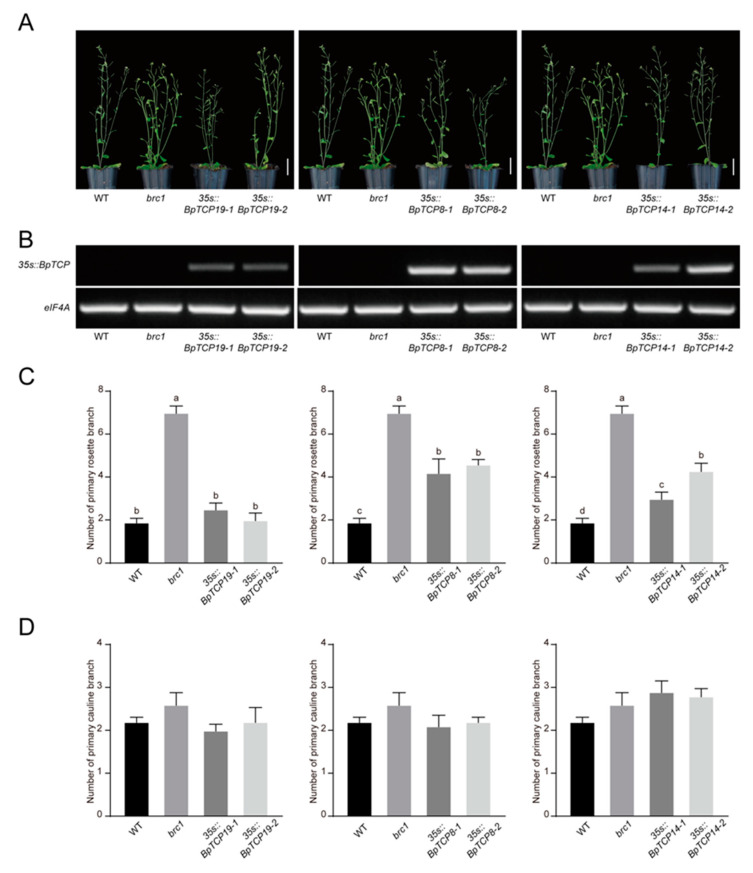
Complementation of *Arabidopsis brc1* mutant phenotype with *BpTCP8*, *BpTCP14* and *BpTCP19*. (**A**) Branching phenotypes of wild-type (WT), *brc1* and representative individuals of two independent *35s::BpTCPs* lines in *brc1* background. Scale bar = 5 cm. (**B**) Expression levels of *BpTCP8*, *BpTCP14* and *BpTCP19* of the seedlings shown in (**A**) were determined by RT-PCR. Expression level of *eIF4A* was detected as a normalization control. (**C**) Number of primary rosette branch of seedlings presented in (**A**). (**D**) Number of primary cauline branch of seedlings presented in (**A**). The number of primary rosette or cauline branch with a length of at least 1 cm was counted. Error bars represent SE (*n* = 10). Different lowercase letters denote significant differences (*p* < 0.05).

**Figure 9:**
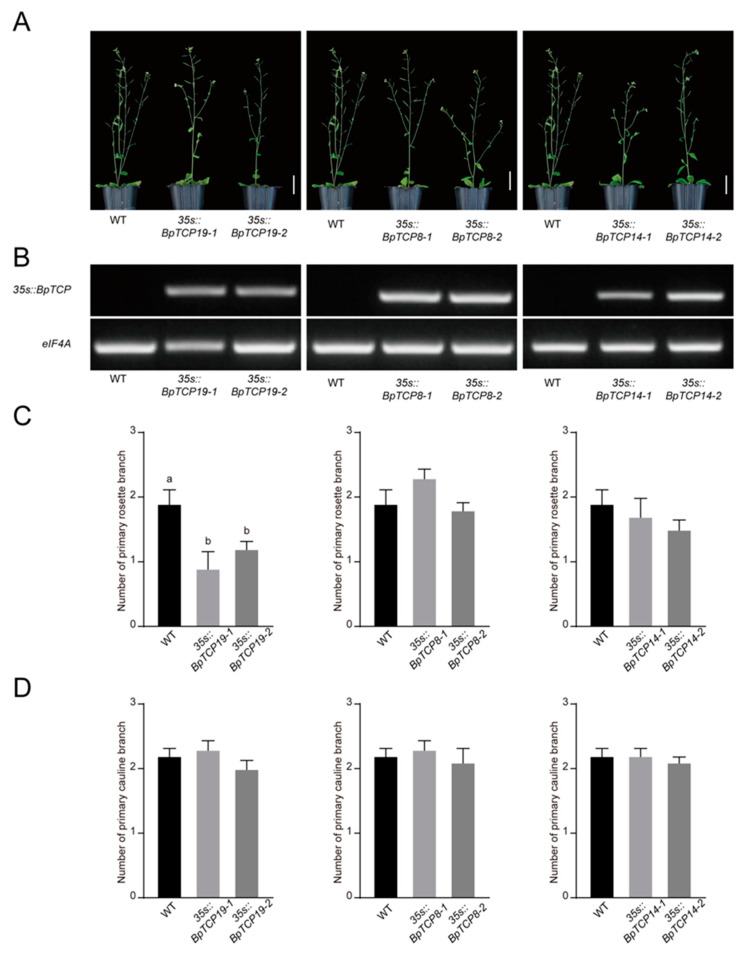
Ectopic expression of *BpTCP8*, *BpTCP14* and *BpTCP19* in *Arabidopsis* wild-type. (**A**) Branching phenotypes of wild-type (WT) and representative individuals of two independent *35s::BpTCPs* lines in wild-type background. Scale bar = 5 cm. (**B**) Expression levels of *BpTCP8*, *BpTCP14* and *BpTCP19* of the seedlings shown in (**A**) were determined by RT-PCR. Expression level of *eIF4A* was detected as a normalization control. (**C**) Number of primary rosette branch of seedlings presented in (**A**). (**D**) Number of primary cauline branch of seedlings presented in (**A**). The number of primary rosette or cauline branch with a length of at least 1 cm was counted. Error bars represent SE (*n* = 10). Different lowercase letters denote significant differences (*p* < 0.05).

To investigate whether the functions of *BpTCP8*, *BpTCP14* and *BpTCP19* are similar with *AtBRC1* in regulating shoot branching, *35s::BpTCPs* were transferred into *Arabidopsis* wild-type and *AtBRC1* ortholog mutant *brc1* by the way of ectopic expression. Two independent transgenic lines of each *BpTCP* gene were selected for phenotypic analysis (Figure 8A and Figure 9A). The expression levels of *BpTCP8*, *BpTCP14* and *BpTCP19* were detected by RT-PCR and all transgenic lines exhibited elevated expression levels (Figure 8B and Figure 9B). Under the same growth conditions, the *brc1* mutants exhibited an apparently luxuriant rosette branches than wild-type plants (Figure 8A). The ectopic expression of *BpTCP8*, *BpTCP14* or *BpTCP19* in *brc1* could reduce the number of primary rosette branches, which was a significant difference from *brc1*. Furthermore, *BpTCP19* was sufficient to restore the number of rosette branches of *brc1* to the wild-type (Figure 8C). In wild-type plants, the ectopic expression of *BpTCP19* could also lead to a decreasing number of primary rosette branches, which was apparently distinguishable from the wild-type, but *BpTCP8* and *BpTCP14* could not (Figure 9C). On the other hand, all of the transgenic plants showed a similar number of primary cauline branches as the *brc1* or the wild-type (Figure 8D and Figure 9D). These results imply that *BpTCP8*, *BpTCP14* and *BpTCP19* prevent primary rosette branch outgrowth.

## 3. Discussion

As a kind of vigorous pioneer woody plant, *B. papyrifera* is widely used in livestock feed, papermaking and ecological afforestation. Genome-wide identification and analysis of the *BpTCP* gene family are of great significance for understanding the development of leaf and branch of *B. papyrifera*. In this study, we carried out a multilevel analysis of *BpTCP* genes with the aim to understand the important and diverse roles involved in the growth and development of *B. papyrifera*.

### 3.1. Overview of BpTCP Gene Family

The analysis of evolution relationship exhibited that 20 BpTCP proteins were classified into three major subclades, which was similar to the previous studies of *A. thaliana*, *O. sativa*, *V. vinifera*, *Z. mays* and *P. vulgaris* [42,43,44,48]. In addition, the composition of conserved motifs in each subclade member showed its particular features. For instance, motif 2, motif 4 and motif 10 were unique to the PCF subclade. All CYC/TB1 subclade members (BpTCP8, BpTCP14 and BpTCP19) and two CIN subclade members (BpTCP10 and BpTCP15) contained motif 6 (that is, R domain), which is similar to that in *VvTCPs*, *PmTCPs* and *FvTCPs* [42,49,50]. R domain is hypothesized to form a hydrophilic α-helix or a coiled coil structure that may mediate protein–protein interactions [1]. The exon/intron organization of *BpTCPs* was most similar to *FvTCPs*, among which only one CYC/TB1 subclade gene had two exons in the coding region and the rest members had one exon [50]. In addition, the number of BpTCP family members was relatively conserved with *A. thaliana* (24), *O. sativa* (21), *Sorghum bicolor* (19), *Medicago truncatula* (21), *Prunus persica* (20) and *M. notabilis* (22), but was smaller than *M. domestica* (58), *Glycine max* (56), *M. acuminate* (45) and *Z. mays* (44) (Figure 2). Combined with genome size, it was found that the number of TCP family members was not related to the genome size. For example, the genome size of *Phyllostachys heterocycla* is 2075 Mb with only 17 TCP members, while the genome size of *M. domestica* is 742.3 Mb with 58 TCP members [51,52]. The diversity of the number of TCP family members in different species may be influenced by genome duplication events, such as whole genome duplication, segmental duplication or tandem duplication. The analysis of duplication events of *BpTCP* genes showed that there were no tandem duplication events, but there were four segment gene pairs. It suggested that segmental duplications may be the primary driving force for the expansion of the *BpTCP* gene family, as described previously, in *V. vinifera* and *Gossypium raimondii* [42,53].

### 3.2. Potential Functions of BpTCP Genes Inferred from the Expression Patterns

The RNA-Seq data of nine tissues (shoot apex, young leaf, developing leaf, mature leaf, immature stem, phloem of proximal stem, phloem of mature stem, phloem of root and root tip) were used to investigate the expression patterns of *BpTCP* genes. According to the expressive level of *BpTCP* genes in specific tissues, the development process in which they probably participate could be speculated. As previously reported, class I (PCF) genes usually promoted cell growth and proliferation [14,54]. In contrast, class II (CIN and CYC/TB1) members had an antagonistic effect on some biological processes compared with class I members. Class II members majorly played a vital role in preventing cell proliferation and tissue overgrowth [55]. CIN subclade genes mainly regulated leaf development. The *cin* mutant showed a phenotype of negative leaf curvature because the regulation of cell division was disturbed [10]. In *Arabidopsis*, the *tcp2 tcp4* mutant exhibited enlarged flat leaves and the quadruple mutant *tcp2 tcp3 tcp4 tcp10* exhibited strongly crinkled leaves [9,29]. CYC/TB1 subclade genes mainly participate in lateral branch and floral symmetry. The *cyc* mutant showed semipeloric flowers and the *tb1* mutant showed excessive axillary branches [2,56]. *AtTCP1* is the ortholog gene of *AmCYC*, which could regulate the longitudinal elongation of the petioles, rosette leaves and inflorescent stems [57]. *AtTCP18* and *AtTCP12*, two homologs of *ZmTB1*, were proved to suppress bud outgrowth [11,34]. In *B. papyrifera*, most *BpTCP* genes from the PCF subclade were relative highly expressed in almost all test samples and less tissue-specific expression patterns, which were similar with *VvTCPs* and *MtTCPs* [42,58]. It implied that *BpTCP* genes of the PCF subclade might be involved in multiple growth and development stages. Two-thirds of CIN subclade genes were abundantly expressed in shoot apex, young leaf, developing leaf and mature leaf. These findings indicated that *BpTCP1*, *BpTCP10*, *BpTCP13* and *BpTCP15* may play an important role in leaf development. In the CYC/TB1 subclade, combined the RNA-Seq and quantitative RT-PCR data, *BpTCP8* and *BpTCP19* were transcribed at relatively high levels in leaf axil and axillary bud. It suggested that *BpTCP8* and *BpTCP19* may regulate the axillary bud outgrowth. Taken together, *BpTCP* genes may play diverse biological functions in organ or tissue development and shoot branching.

### 3.3. BpTCP8, BpTCP14 and BpTCP19 Prevent Branch Outgrowth

It was shown that the *BRC1* gene was the focal points for multiple environmental and developmental signals that acted in the axillary buds to inhibit shoot branching. The homologs of *AtBRC1* in tomato, pea, chrysanthemum, poplar and cucumber could inhibit lateral bud or branch outgrowth [59,60,61,62,63]. In chrysanthemum, *DgBRC1s* were mainly expressed in the nodes containing axillary buds and expression of *DgBRC1s* in *Arabidopsis brc1* or wild-type could reduce the number of rosette branches [61]. In woody plant poplar, the loss-of-function mutant of *PcBRC1* exhibited strongly enhanced bud outgrowth. The mutant of *PcBRC2* showed an extreme bud outgrowth and had two ectopic leaves at each node, which was different with *Arabidopsis*. *PcBRC2* may have retained or evolved the function of controlling leaf development [62]. In our work, we validated the function of *BpTCP8*, *BpTCP14* and *BpTCP19* on shoot branching in both *Arabidopsis* wild-type and *brc1* mutant backgrounds. The functions of *BpTCP8*, *BpTCP14* and *BpTCP19* on inhibiting rosette branch outgrowth in *Arabidopsis* was similar to chrysanthemum [61]. In addition, the difference of our study from previous studies is that all *BpTCP* genes (*BpTCP8*, *BpTCP14* and *BpTCP19*) in the CYC/TB1 subclade possessed the ability to suppress rosette branch outgrowth in *Arabidopsis*, not just the ortholog genes of *AtBRC1*. The analysis of duplication events showed that these three genes are involved in two segmental duplication events (*BpTCP8*/ *BpTCP14* and *BpTCP8*/ *BpTCP19*), which may be the reason for the functional redundancy in regulating the branch outgrowth. The CYC/TB1 subclade genes are considered to regulate shoot branching and floral symmetry [64]. Therefore, *BpTCP8*, *BpTCP14* and *BpTCP19* could prevent lateral branch growth, same as shown in previous research, and may participate in leaf development like that of poplar.

## 4. Materials and Methods

### 4.1. Identification of Putative BpTCP Genes in B. papyrifera

To identify the TCP family in *B. papyrifera* genome, two approaches were used. Local BLASTp searches were performed against the *B. papyrifera* genome database using the 24 known *Arabidopsis* TCP protein sequences, setting the threshold of the E-value to 1 × 10^−5^ for the initial identification. Additionally, we downloaded the hidden Markov model (HMM) seed file of TCP domain (PF03634) from the Pfam database (http://pfam.xfam.org/), and used the HMMER software to carry out the HMM searches against the local *B. papyrifera* protein sequence database, setting the threshold of the E-value to 0.01. Summarizing the results of both methods and removing the redundant sequences, the remaining sequences were the candidate TCP protein sequences of *B. papyrifera*. Subsequently, all candidate BpTCP protein sequences were submitted to the NCBI Conserved Domain Search Service (CD Search) (https://www.ncbi.nlm.nih.gov/Structure/cdd/wrpsb.cgi) [65] and InterPro (http://www.ebi.ac.uk/interpro/) database for the further verification. The *Arabidopsis* TCP proteins were downloaded from The Arabidopsis Information Resource (TAIR) database (http://www.arabidopsis.org). The molecular weight (MW) and isoelectric point (pI) of each BpTCP protein was predicted by the ExPASy program (https://web.expasy.org/protparam/). The subcellular localization of each BpTCP protein was predicted by the CELLO server (http://cello.life.nctu.edu.tw/). The online tool P3DB (http://www.p3db.org/) was used to carry out the phosphorylation analysis.

### 4.2. Phylogenetic Analysis

For phylogenetic analysis, the full-length amino acid sequences of TCP proteins from *Arabidopsis*, *O. sativa*, *B. papyrifera*, *Z. mays* (ZmTB1) and *A. majus* (AmCYC, AmCIN) were aligned by MEGA X software with default parameters [66]. The phylogenetic tree was constructed by the maximum likelihood (ML) method with 1000 bootstrap replicates. The JTT model was selected as the optimal model. The *O. sativa* TCP protein sequences were retrieved from the PlantTFDB (http://planttfdb.cbi.pku.edu.cn/). The sequences of ZmTB1, AmCYC and AmCIN were downloaded from the Phytozome database v12.1 (https://phytozome.jgi.doe.gov/pz/portal.html).

### 4.3. Gene Structure and Conserved Motif Analysis

The gene structure of *BpTCP* genes was analyzed by the online website GSDS 2.0 (http://gsds.cbi.pku.edu.cn). The conserved motif was predicted by the Multiple Em for Motif Elicitation (MEME) server (http://meme-suite.org/), and the program was performed with the following settings: site distribution = any number of repetitions; 0-order model of sequences for the background motif; motif width = 15–60 and maximum number of motifs = 10.

### 4.4. Chromosomal Localization and Duplication Event Analysis

The chromosomal location information of all *BpTCP* genes was obtained from the *B. papyrifera* genome database. The visualized diagram of chromosomal localization and the duplication events in the *B. papyrifera* were acquired from the TBtools software [67].

### 4.5. Expression Analysis of BpTCP Genes in Different Tissues

Expression level of 20 *BpTCPs* in different tissues was detected by an Illumina HiSeq 2500 platform. The shoot apex, young leaf, developing leaf, mature leaf, immature stem, phloem of proximal stem, phloem of mature stem, phloem of root and root tip were collected from a five-year-old female *B. papyrifera* and immediately frozen in liquid nitrogen. The total RNA was isolated and purified to construct the RNA-Seq libraries, then sequenced. Each tissue had three biological replicates. Gene expression was measured as fragments per kilobase of transcript per million fragments mapped (FPKM) using Cuffquan and CuffnormGene in Cufflinks. The expression values of each gene in different tissues were averaged and presented as a log value. The heat map was exhibited using the TBtools software [67]. The FPKM value expressed in different tissues are listed in Table S2.

### 4.6. Plant Materials and Growth Conditions

*Arabidopsis* ecotype Columbia-0 (Col-0) was used as the wild-type. The mutant *brc1* was described previously [11]. Murashige and Skoog medium with or without 25 mg/mL hygromycin B was used for screening or growing the plant seeds. The plates with seeds were put at 4 °C for two days for synchronization before cultivation at 22 °C under long-day conditions with a photoperiod of 16 h/8 h (light/dark), photosynthetic photon flux density of 200 μmol m^−2^s^−1^ and 60% relative humidity. The culture matrix was a mixture of vegetative soil and vermiculite with a ratio of 2:1.

The plantlets of *B. papyrifera* were cultured on the MS medium at 26 °C with a photoperiod of 14 h/10 h (light/dark) and photosynthetic photon flux density of 80 μmol m^−2^s^−1^. A month later, the shoot apex, leaf axil, axillary bud, leaf, stem and root were collected for quantitative RT-PCR.

### 4.7. Quantitative RT-PCR

Total RNA was extracted from different tissues using the TransZol Plant Mini kit (TransGen, Beijing, China) according to the manufacturer’s instructions. The first-strand cDNA was synthesized by using a PrimeScript^TM^ RT reagent kit with gDNA eraser (Takara, Dalian, China). The quantitative RT-PCR reactions were carried out on a StepOne^TM^ real-time PCR system (Applied Biosystems, Forster City, USA) using the SYBR^®^ Premix Ex Taq^TM^ II kit (Takara, Dalian, China). Each reaction was operated in a 20 μL volume, which contained 2 μL diluted cDNA, 0.4 μL forward/reverse primer (10 μmol/L), 6.8 μL sterilized ddH_2_O, 0.4 μL ROX Reference Dye II and 10 μL SYBR^®^ Premix Ex Taq II. Three technical replicates were performed for each biological sample. The Glyceraldehyde-3-phosphate Dehydrogenase (*GAPDH*) gene of *B. papyrifera* was used as an internal control. The 2^−∆∆Ct^ method was used to calculate the data. The primer sequences of each gene involved in quantitative RT-PCR are listed in Table S3.

### 4.8. Transcriptional Activation Activity Analysis

The coding region of the *BpTCP8*, *BpTCP14* and *BpTCP19* was amplified and cloned into the *p*Bridge vector to generate a fusion protein with the GAL4 DNA binding domain, respectively. The fusion constructs and control vector were transferred into the AH109 yeast strain. Then the transformed yeast cells were dropped on SD-Trp-His medium with 0–50 mM 3-AT to detect their activities. The plates were cultured at 30 °C for 3–5 days before photographing.

### 4.9. Vector Construction and Plant Transformation

The coding region of the *BpTCP8*, *BpTCP14* and *BpTCP19* was inserted into the *p*CAMBIA1300 vector containing a cauliflower mosaic virus (CaMV) 35S promoter. The constructs were then transformed into the *Agrobacterium tumefaciens* strain EHA105. Both wild-type and *brc1* plants were transformed by the floral dip method. In addition, transgenic plants were selected on the MS medium with 25 mg/mL hygromycin B for one week. Homozygous plants were used for the branching phenotype analysis in this study. The eukaryotic initiation factor 4A (*eIF4A*) gene of *Arabidopsis* was used as a normalization control. Primers used for semi-quantitative are listed in Table S3.

## 5. Conclusions

In this study, we identified 20 *BpTCP* genes, which distributed on 10 chromosomes. These BpTCPs were divided into three subclades according to the phylogenetic relationships and structural properties. Segmental duplication was the predominant duplication event, which induced the expansion of *BpTCP* genes. Expression patterns in different tissues suggested that *BpTCP* genes may play important roles in *B. papyrifera* growth and development. BpTCP8, BpTCP14 and BpTCP19 were verified that they possessed transcriptional activation ability, indicating that they were functional transcription factors. In addition, the function of *BpTCP8*, *BpTCP14* and *BpTCP19* in the regulation of shoot branching was confirmed. Ultimately, these findings will provide a solid foundation for the further functional investigation of *BpTCP* genes and the improvement of new cultivars via genetic engineering.

## Figures and Tables

**Table 1:** *TCP* genes in *B. papyrifera.*

Gene Name	Gene ID	Protein			Subcellular Localization	No. of Phosphorylation Site		
		Length(aa)	pI	MW(Da)		Ser Site	Thr Site	Tyr Site
*BpTCP1*	Bp01g1625.1	431	6.42	45,631.72	Nuclear	5	1	0
*BpTCP2*	Bp01g1872.1	382	9.35	40,857.4	Nuclear	4	0	0
*BpTCP3*	Bp03g0503.1	372	5.31	38,695.22	Nuclear	3	1	0
*BpTCP4*	Bp04g1763.1	213	8.88	22,559.33	Nuclear	4	0	0
*BpTCP5*	Bp06g0134.1	410	5.6	42,674.86	Nuclear	4	3	0
*BpTCP6*	Bp07g0091.1	268	9.37	28,552.82	Nuclear	4	0	0
*BpTCP7*	Bp07g0581.1	435	7.3	45,677.16	Nuclear	6	2	0
*BpTCP8*	Bp07g0899.1	456	6.86	50,701.91	Nuclear	8	1	0
*BpTCP9*	Bp08g1476.1	411	5.26	45,966.93	Nuclear	0	0	0
*BpTCP10*	Bp08g1806.1	494	7.9	53,033.04	Nuclear	3	1	0
*BpTCP11*	Bp09g1510.1	165	5.27	18,140.32	Nuclear	0	0	0
*BpTCP12*	Bp09g2064.1	566	6.91	59,824.02	Nuclear	2	1	0
*BpTCP13*	Bp10g0666.1	365	6.3	38,782.52	Nuclear	1	1	0
*BpTCP14*	Bp10g1127.1	413	9.02	46,005.71	Nuclear	3	2	0
*BpTCP15*	Bp10g1216.1	390	6.27	42,962.47	Nuclear	0	0	1
*BpTCP16*	Bp10g1801.1	343	9.08	35,185.94	Nuclear	3	0	0
*BpTCP17*	Bp12g0868.1	433	6.99	45,893.42	Nuclear	6	1	0
*BpTCP18*	Bp12g1015.1	331	9.15	36,853.02	Nuclear	1	1	3
*BpTCP19*	Bp12g1157.1	444	9.06	48,894.63	Nuclear	5	0	0
*BpTCP20*	Bp13g0172.1	277	9.72	28,469.7	Nuclear	2	0	0

MW: molecular weight; pI: isoelectric point.

## Accession Number

Sequence data from this article can be found in the NCBI database under Sequence Read Archive (SRA) ID SRP136442.

## Supplementary Materials

The following are available online at https://www.mdpi.com/2223-7747/9/10/1301/s1, Figure S1: Chromosomal distribution of BpTCP genes, Figure S2: The sequence logo of conserved motifs in BpTCP proteins, Table S1: Distribution of TCP proteins in plant, Table S2: FPKM value expressed in different tissues, Table S3: Primers of quantitative and semi-quantitative RT-PCR.
