# Supplementary material for: Genome-Wide Identification of the TCP Gene Family in Broussonetia papyrifera and Functional Analysis of BpTCP8, 14 and 19 in Shoot Branching
Source: Plants (Basel). 2020 Oct 1;9(10):1301. doi: 10.3390/plants9101301 (PMC7650637; doi:10.3390/plants9101301)
Supplement: Supplementary file 1 [file plants-09-01301-s001.zip › Supplementary Materials.docx]

Supplementary Materials


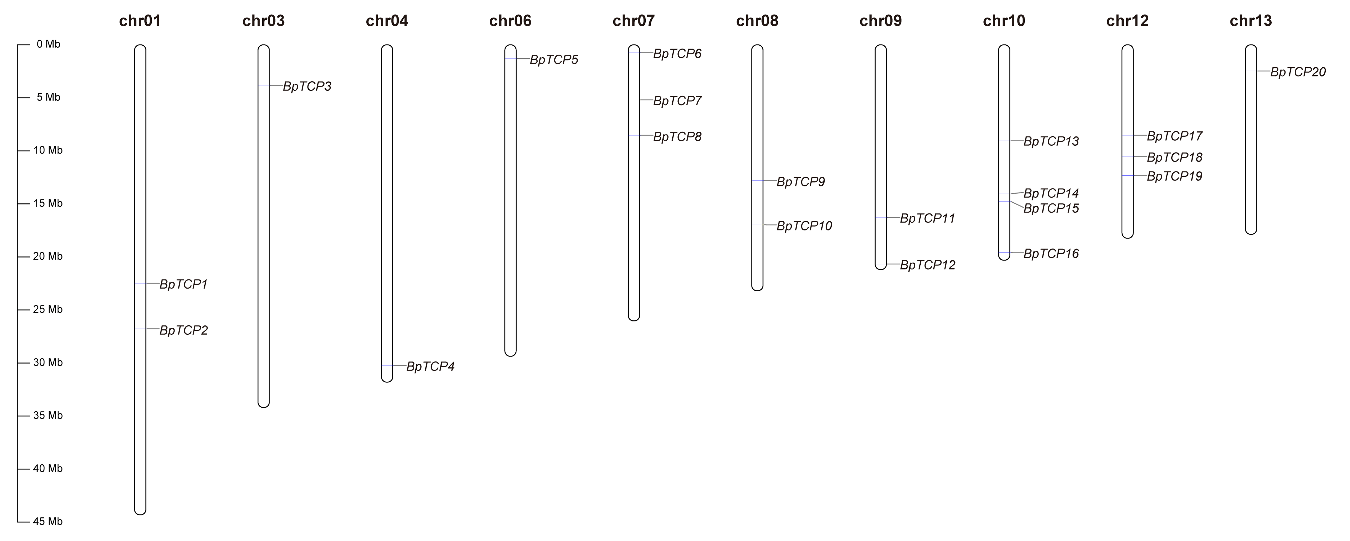


**Figure S1.** Chromosomal distribution of BpTCP genes.


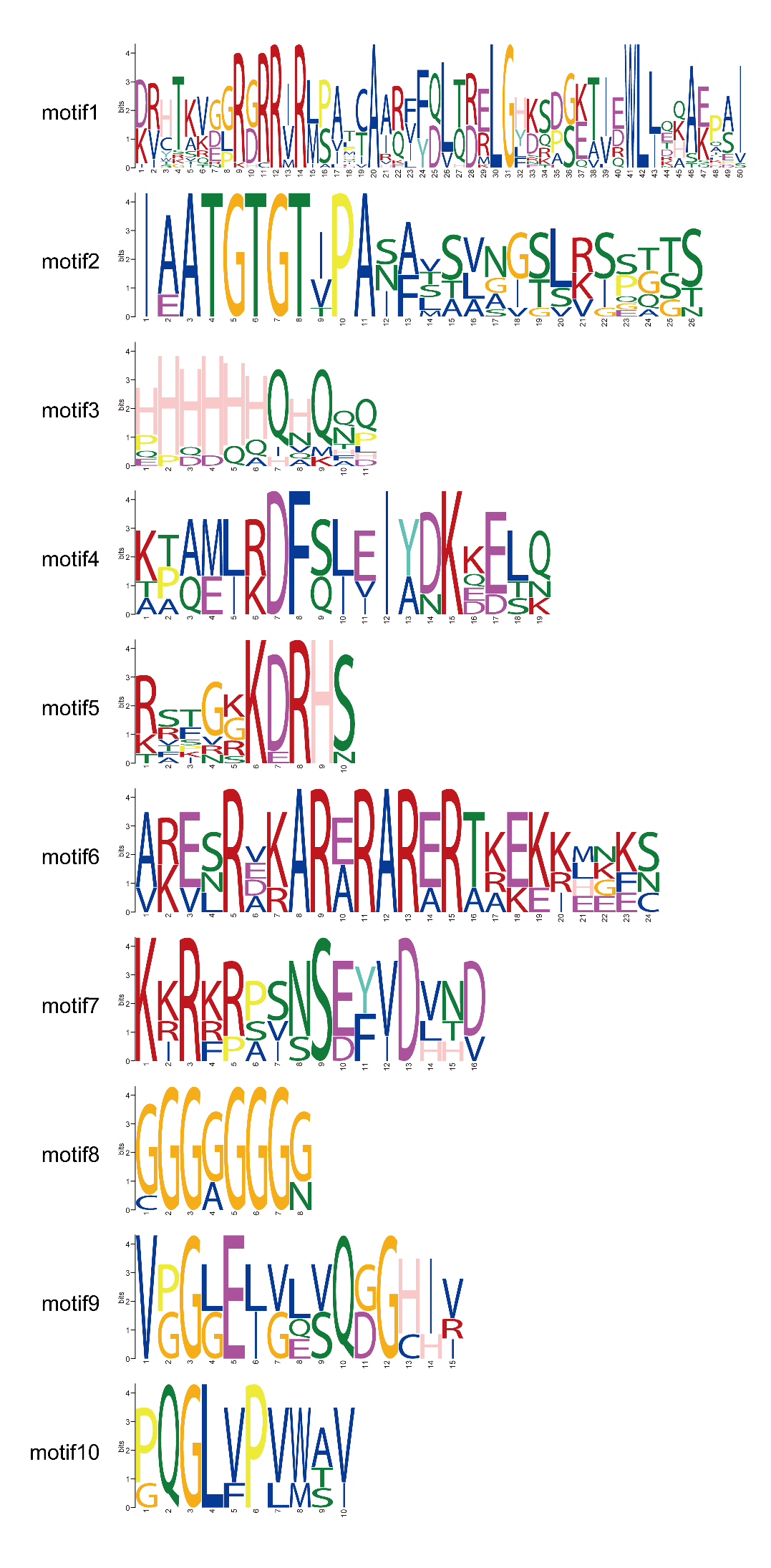


**Figure S2.** The sequence logo of conserved motifs in BpTCP proteins.

**Table S1.** Distribution of TCP proteins in plant.

| **Plants** | **Number of TCP** | **Reference** |
| --- | --- | --- |
| *Brachypodium distachyon* | 21 | <http://planttfdb.cbi.pku.edu.cn/> |
| *Phyllostachys heterocycla* | 17 | <http://planttfdb.cbi.pku.edu.cn/> |
| *Zea mays* | 44 | <http://planttfdb.cbi.pku.edu.cn/> |
| *Sorghum bicolor* | 19 | <http://planttfdb.cbi.pku.edu.cn/> |
| *Oryza sativa* | 21 | <http://planttfdb.cbi.pku.edu.cn/> |
| *Musa acuminata* | 45 | <http://planttfdb.cbi.pku.edu.cn/> |
| *Aquilegia coerulea* | 16 | <http://planttfdb.cbi.pku.edu.cn/> |
| *Vitis vinifera* | 15 | <http://planttfdb.cbi.pku.edu.cn/> |
| *Glycine max* | 56 | <http://planttfdb.cbi.pku.edu.cn/> |
| *Medicago truncatula* | 21 | <http://planttfdb.cbi.pku.edu.cn/> |
| *Prunus persica* | 20 | <http://planttfdb.cbi.pku.edu.cn/> |
| *Fragaria vesca* | 18 | <http://planttfdb.cbi.pku.edu.cn/> |
| *Malus domestica* | 58 | <http://planttfdb.cbi.pku.edu.cn/> |
| *Broussonetia papyrifera* | 20 | <http://planttfdb.cbi.pku.edu.cn/> |
| *Morus notabilis* | 22 | <http://planttfdb.cbi.pku.edu.cn/> |
| *Populus trichocarpa* | 37 | <http://planttfdb.cbi.pku.edu.cn/> |
| *Eucalyptus grandis* | 16 | <http://planttfdb.cbi.pku.edu.cn/> |
| *Arabidopsis thaliana* | 24 | <http://planttfdb.cbi.pku.edu.cn/> |
| *Theobroma cacao* | 21 | <http://planttfdb.cbi.pku.edu.cn/> |
| *Gossypium raimondii* | 38 | <http://planttfdb.cbi.pku.edu.cn/> |
| *Petunia axillaris* | 28 | <http://planttfdb.cbi.pku.edu.cn/> |
| *Solanum lycopersicum* | 36 | <http://planttfdb.cbi.pku.edu.cn/> |
| *Antirrhinum majus* | 30 | Li et al., 2019 |
| *Amborella trichopoda* | 15 | <http://planttfdb.cbi.pku.edu.cn/> |
| *Selaginella moellendorffii* | 6 | <http://planttfdb.cbi.pku.edu.cn/> |
| *Physcomitrella patens* | 7 | <http://planttfdb.cbi.pku.edu.cn/> |
| *Marchantia polymorpha* | 2 | <http://planttfdb.cbi.pku.edu.cn/> |
| *Volvox carteri* | 0 | <http://planttfdb.cbi.pku.edu.cn/> |
| *Dunaliella salina* | 0 | <http://planttfdb.cbi.pku.edu.cn/> |
| *Chlamydomonas reinhardtii* | 0 | <http://planttfdb.cbi.pku.edu.cn/> |
|  |  |  |
| Li, M., Zhang, D., Gao, Q., Luo, Y., Zhang, H., Ma, B., Chen, C., Whibley, A., Zhang, Y.e., Cao, Y., Li, Q., Guo, H., Li, J., Song, Y., Zhang, Y., Copsey, L., Li, Y., Li, X., Qi, M., Wang, J., Chen, Y., Wang, D., Zhao, J., Liu, G., Wu, B., Yu, L., Xu, C., Li, J., Zhao, S., Zhang, Y., Hu, S., Liang, C., Yin, Y., Coen, E. and Xue, Y., 2019. Genome structure and evolution of Antirrhinum majus L. Nat. Plants 5, 174-183. | | |
|  |  |  |

**Table S2.** FPKM value expressed in different tissues.

(See in attachment [Table S2.xlsx](Table%20S2.xlsx))

**Table S3.** Primers of quantitative and semi-quantitative RT-PCR.

| **Gene names** | **Forward primer** | **Reverse primer** | **Purpose** |
| --- | --- | --- | --- |
| *BpTCP8* | ATTCTGGAGAAATCGCGGCA | AAACTGATCCTTGAGTTCCTCTTGT | quantitative |
|  | GCTCGGATTTGAGAGGGCAA | GTAGAGCCTGCAACAAAGCC | semi-quantitative |
| *BpTCP14* | AGGGCCAGGGCGAGG | CTCTTCTTGATCACGATCACCAGT | quantitative |
|  | CCCACTTGAGAACACCTCCG | ACTTGGTGATGAGCCACTCG | semi-quantitative |
| *BpTCP19* | ACCAGAGAGGCTACGGACAT | TCACACTGCATCTGGTCAGC | quantitative |
|  | AGACCGCCACAGCAAGATTT | AACCGCATTGCCCCACATTA | semi-quantitative |
| *GAPDH* | TCAACATCATTCCTAGCAGTACCG | AGTCAGTGGAAACCACGTCATC | quantitative |
| *eIF4A* | GGGTATCTATGCTTACGGTTTCG | CAGAGAACACTCCAACCTGAATC | semi-quantitative |
